# Superior Facet Joint Violations during Single Level Minimally Invasive Transforaminal Lumbar Interbody Fusion: A Preliminary Retrospective Clinical Study

**DOI:** 10.1155/2018/6152769

**Published:** 2018-03-05

**Authors:** Long Jia, Yan Yu, Kamran Khan, Fuping Li, Rui Zhu, Zhili Zeng, Liming Cheng

**Affiliations:** ^1^Department of Spine Surgery, Tongji Hospital, Tongji University School of Medicine, Shanghai 200065, China; ^2^Department of Orthopedics, Zhongshan Hospital Affiliated to Fudan University, Qingpu Branch, Shanghai 201700, China; ^3^Department of Orthopedic Surgery, Massachusetts General Hospital, Harvard Medical School, Boston, MA 02114, USA; ^4^Department of Histology and Embryology, Tongji University School of Medicine, Shanghai 200092, China

## Abstract

**Background:**

Facet joint violation (FV) was reported as variable iatrogenic damage that can be a crucial risk factor leading to the adjacent segment degeneration (ASD). “Blind” screw placement technique in minimally invasive transforaminal lumbar interbody fusion (MIS-TLIF) contributes to the increasing incidence of FV that can be influenced by several potential factors. Many controversies about these factors and clinical outcomes of different types of FV patients exist, yet they have not been analyzed.

**Methods:**

99 cases undergoing single-segment MIS-TLIF from July 2013 to December 2015 were retrospectively analyzed. Computed tomography (CT) was applied to determine the incidence of FV, and then the correlation between FV and relevant factors, including gender, age, body mass index (BMI), top-screw level, and decompression, was analyzed. A total of 53 cases were followed up after one year, 31 cases in noninjury (A group) and 22 patients in FV injury (B group).

**Results:**

The incidence of FV was 39. 39% (39/99) in the patients and 23.23% (46/198) in the screws. Logistic regression analysis showed that screw at L5 in patients with BMI > 30 kg/m^2^ was vulnerable to FV (*P* < 0.05). Moreover, postoperative average intervertebral disc height (AIDH) of fusion segment, visual analog scale (VAS), and Oswestry disability index (ODI) scores improved significantly in group A and B when compared with preoperative data (*P* < 0.05). Adjacent superior average intervertebral disc height (ASAIDH) presented decrease, but adjacent superior intervertebral disc Cobb angle (ASIDCA) appeared to increase in the two groups at the final follow-up compared with postoperative 3 days (*P* < 0.05). Low back VAS and ODI scores in group A (31 cases) were lower than those in group B (22 cases) in the final follow-up (*P* < 0.05).

**Conclusion:**

MIS-TLIF is an effective treatment for lumbar degenerative disease, but FV occurred at a higher incidence. Facet joints should be protected in MIS-TLIF to avoid FV.

## 1. Introduction

The traditional open transforaminal lumbar interbody fusion (TLIF) is successful in treating various symptomatic spinal conditions, but complications like extensive blood loss and prolonged recovery time continue to remain a major concern [1]. To address these issues, minimally invasive transforaminal lumbar interbody fusion surgery (MIS-TLIF) has become a popular alternative. The reported advantages of MIS-TLIF include faster recovery time, less blood loss, improved postoperative pain and lower healthcare cost, all due to minimal soft-tissue injury [2, 3].

One major concern for MIS-TLIF is that it relies on fluoroscopic guidance rather than direct visualization of anatomic landmarks to insert pedicle screws. This loss of visualization increases the risk of facet join violation (FV) at the superior motion segment [4–6]. Injury to the superior facet joints during placement of pedicle screws causes facet joint stiffness, rigidity, osteoarthritis and accelerates adjacent segment disease (ASD) [6]. Moreover, FV can contribute to increased stresses at the adjacent segment leading to biomechanical changes like abnormal facet joint loading and aberrant motion which can accelerate ASD. The rate of symptomatic ASD at an adjacent segment requiring a reoperation is 36.1% at ten years [7]. This illustrates the urgent need to avoid adjacent FV to decrease healthcare spending by reducing reoperations and improving patient safety and quality of life.

Several studies have reported the risk factors such as age, sex, weight, top-screw level in FV in percutaneous pedicle screw placement [8–11]. However, there is a lack of consensus on the etiology of FV, mainly due to mixed findings from studies. A possible explanation for mixed findings is that the studies did not choose proper controls and had overlapping patient groups and a short follow-up time. Moreover, clinical outcomes of different levels of FV in patients undergoing MIS-TLIF have not been analyzed. In addition, predictors of ASD like reduced disc height and abnormal Cobb angle have not been studied in conjunction with clinical outcomes for different FV in MIS-TLIF. Therefore, in this study, we retrospectively analyzed 99 cases of patients who underwent one-level MIS-TLIF. The incidence of FV, relevant risk factors in FV resulting from adjacent superior pedicle screw insertion, type of FV, adjacent segment disc height, and Cobb angle were recorded at preoperation and at 1-year follow-up. These factors were then compared with patient outcomes at 1 year postoperation to observe a possible correlation. Comprehensive understanding of the factors involved in different levels of FV and how they are correlated with clinical outcomes is a critical component in avoiding revision surgery for symptomatic ASD and this study aims to support that knowledge gap.

## 2. Materials and Methods

This study was reviewed and approved by the Human Ethics Committee of the Tongji Hospital, Tongji University School of Medicine, China. This was a retrospective study, so individual consent was waived.

### 2.1. Patient Cohort

A cohort of 99 adult patients (51 male, 48 female, average age: 54.57 ± 12.44 years old) who underwent one-level MIS-TLIF at the Tongji Hospital between July 2013 and December 2015 were identified via electronic medical records. Inclusion criteria were chosen based on the minimally invasive lumbar fusion guidelines. Patients with lumbar spondylolysis, instability, and spondylolisthesis (*I*°) were included. The chosen patient cohort had top-screws at L4 in 66 cases and at L5 in 33 cases. Patients with revision surgery, spinal tumors, degenerative lumbar scoliosis, and other spinal conditions were excluded. All patients were diagnosed as having low back and leg pain without relief following a conservative treatment for more than 3 months.

Computed tomography (CT) and magnetic resonance imaging (MRI) were used to classify lumbar disc herniation, lumbar spinal stenosis, and spondylolisthesis. All operations were performed by 3 experienced surgeons who had over 10 years' experience in spine surgery.

Radiographic evaluation of top level screws, facet grade, adjacent segment disc height, Cobb angle, and other parameters was made by 2 different radiologists and spine surgeons for an accurate analysis. Shah's classification was used to identify FVs in patients at 1-year follow-up [12]. Patients were grouped according to FV with group A being the noninjury group (bilateral pedicle screws did not violate adjacent superior facet joint). Group B was the injury group and was defined as at least one pedicle screw violating adjacent superior facet joint (Table 1).

### 2.2. Surgical Procedures

Patients were kept in prone position following general anesthesia and their abdomen was suspended and pressure parts were with pad. C-arm fluoroscopy was used to determine available surgical space. A 2 to 3 cm incision was made approximately 2.5 cm lateral to the midline to cut skin and muscular fasciae. After inserting the dilators step by step, Pipeline working channel (Johnson & Johnson Company, NY, USA) was placed into and fixed by dilators, or directly using Spotlight working channel (Johnson & Johnson Company, NY, USA). Then the local soft tissue was removed to expose vertebral plate edges and facet joint. The decompression was performed to expose dural sac, the central canal, lateral crypt, and nerve root canal after removing part of vertebral plate, ligamentum flavum, and facet joints. After thoroughly removing intervertebral disc and cartilage endplate, local autologous bone was implanted into intervertebral space, and then single suitable height of intervertebral fusion was placed. For bilateral decompression or more, the same method was performed to deal with the contralateral and other spaces. Under the guidance of C-arm fluoroscopy, placement of percutaneous pedicle screws was performed using Viper 2 system (Johnson & Johnson Company, NY, USA), and percutaneous rod was also placed using the instruments and prelocked. Drainage tube was removed 24–36 h postoperatively. At 3 days postoperatively, the patients were examined with lumbar X-ray and CT to confirm the position of lumbar fusion instruments and internal fixation and to evaluate the facet joint violation. Patients were encouraged to have activities out of bed under waist protection. Waist torsion and bending activities were prohibited within 3 months under waist protection.

### 2.3. Evaluation Methods

**Figure 1:**
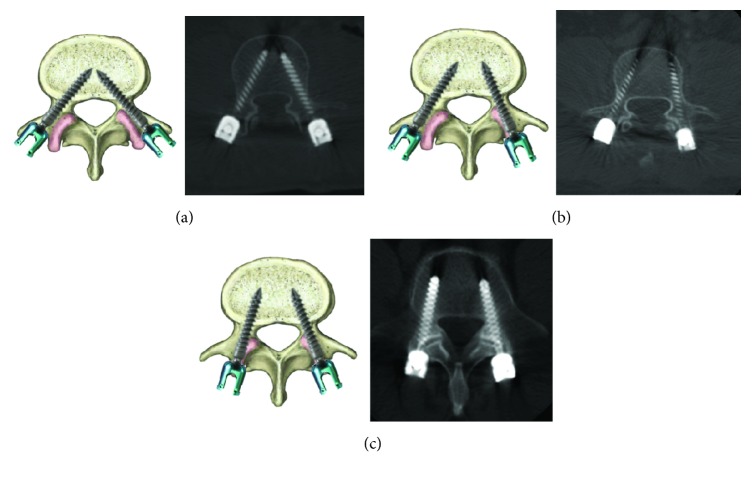
Shah's classification. (a) (grade 1): no facet joint violation; (b) (grade 2): unilateral facet joint violation (screw head contacted or invaded facet joint); (c) (grade 3): bilateral facet joint violation (screw head contacted or invaded facet joint).

High-resolution CT scan with sagittal and coronal reconstructive images were adjusted parallel to the pedicle screws to evaluate the positions in all patients. The facet joint violations were examined using Shah's classification (3 grades, Figure 1) [12]: grade 1, no facet joint violation; grade 2, unilateral facet joint violation (screw head contacted or invaded facet joint); grade 3, bilateral facet joint violation (screw head contacted or invaded facet joint). Two of the authors reviewed the CT images independently to determine the violation status of the facet joints. Association of FV with age (statutory retirement age is 60 years old in China), gender, BMI (World Health Organization defines a BMI of 30 kg/m^2^ or above as obese), top-screw level, and decompression method was analyzed.

**Figure 2:**
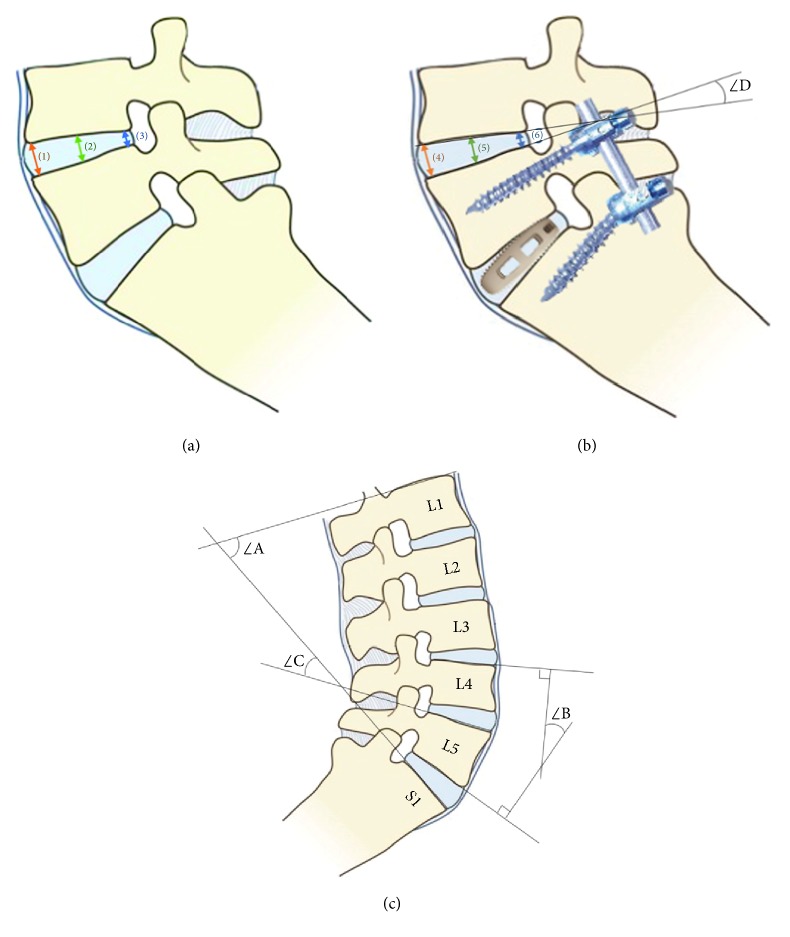
Radiographic method. (a) Average intervertebral disc height (AIDH): (1) anterior of intervertebral disc height, (2) middle of intervertebral disc height, (3) the posterior of intervertebral disc height, AIDH: ((1) + (2) + (3))/3; (b) adjacent superior average intervertebral disc height (ASAIDH) and adjacent superior intervertebral disc Cobb angle (ASIDCA): (4) anterior of adjacent superior intervertebral disc height, (5) middle of adjacent superior intervertebral disc height, (6) posterior of adjacent superior intervertebral disc height, ASAIDH: ((4) + (5) +(6))/3, ∠D for ASIDCA: angle of upper and low endplate; (c) lumbar sagittal curvature: ∠A represents lumbar Cobb: angle of L1 upper endplate and S1 upper endplate, ∠B for the L4/5 Cobb: angle of L4 upper endplate and L5 low endplate, ∠C for L5/S1 Cobb: angle of L5 upper endplate and S1 upper endplate.

Clinical data were collected to compare therapeutic efficacy among different FV patients. Operation duration, blood loss, and postoperative drainage were recorded. Average intervertebral disc height and lumbar and surgical Cobb angle were measured in X-rays before and after operation to assess the recovery of intervertebral space height and the variation of lumbar spine kyphosis (Figure 2). Adjacent superior average intervertebral disc height and adjacent superior intervertebral disc Cobb angle were measured by X-rays 3 days after operation and at 1-year follow-up to assess the gradation of adjacent segment degeneration (Figure 2). Visual analog scale (VAS) score of low back and leg pain and Oswestry disability index (ODI) score were also recorded to assess functional improvement.

### 2.4. Statistical Analysis

All data were expressed as means ± standard deviation. Chi-squared analysis was carried out to determine the association between superior facet joint violation and technical factors. Logistics regression analysis was used to examine simultaneous relationships of violation's risk. Student's *t*-test was used to compare clinical efficacy of different FV patients (such as Adjacent Disc Height, VAS, ODI, Cobb angle). *P* < 0.05 was defined as statistically significant.

## 3. Results

### 3.1. Superior Facet Joint Violation in MIS-TLIF

99 cases (total 198 top-screws) were operated successfully. Postoperative CT scans showed that the level of FV included 60 cases with grade 1 FV (60.61%), 32 cases with grade 2 FV (32.32%), and 7 cases with grade 3 FV (7.07%), and the incidence of FV in MIS-TLIF was 39.39% (39/99) of the patients and 23.23% (46/198) of the screws. Chi-square test showed that screw pedicle placement at L5 and patients with BMI > 30 kg/m2 had a higher prevalence rate of FV (Table 2), while logistic regression analysis also showed that FV was closely related to BMI (odds ratio = 6.451) and top-screw level (odds ratio = 4.668).

### 3.2. Clinical Outcomes of Different Types of FV Patients in MIS-TLIF

At 1-year follow-up, 59 cases with noninjury were assigned as group A. The average operative time was 211.61 ± 43.19 min, average blood loss was 177.42 ± 66.88 ml, and postoperative drainage was 87.10 ± 75.93 ml in group A. Group B had 40 cases with injury and the average operative time was 216.82 ± 45.11 min, average blood loss was 193.18 ± 79.13 ml, and average postoperative drainage was 62.05 ± 54.81 ml. There was no statistical difference in aforementioned factors between the two groups. The intervertebral disc height in the two groups recovered significantly after operation (*P* < 0.05, Table 3), when compared to the preoperative values. VAS and ODI scores for low back and leg at 2 weeks postoperation and at 1-year follow-up significantly improved (*P* < 0.05, Table 3). Adjacent segment average disc height decreased at 1-year follow-up when compared to 3 days' postoperative values in both groups. However, adjacent superior intervertebral disc Cobb angle increased at 1-year follow-up when compared to 3 days' postoperative values in both groups (*P* < 0.05, Table 3). Lastly, low back VAS and ODI scores in group A were lower than those in group B at the final follow-up (*P* < 0.05, Table 3).

## 4. Discussion

Spinal fusion technique is the gold standard in treating spinal disorders but postoperation complications still remain a significant concern for surgeons. The intervertebral fusion technique alters the disc biomechanical environment, thus altering the motion and function of the index and adjacent segments. Specifically, biomechanical studies reveal increased intradiscal pressure, increased tensile and shearing stresses, and increased facet loads, accelerating degenerative changes [13]. ASD is most commonly found in the superior segments [5, 14], especially in adjacent superior segments [15, 16]. After a lumbar fusion surgery follow-up in 1069 cases for more than 10 years, Lee et al. [5] found that degeneration of facet joint played a critical role in the progression of ASD. Transpedicular screw fixation could increase the risk of facet joint violation, which can increase the incidence of ASD [6]. By simulating pedicle screw damage to adjacent superior facet joints using finite element method, Kim et al. showed that the contact stress of facet joint and disc pressure would increase when facet joint violation occurred, while the contact stress of facet joint and disc pressure would decrease when the internal fixation in the injured facet joint was taken out [17]. The duration of the follow-up in this study was not so long, yet the results showed that the average intervertebral disc height decreased and Cobb angle increased at adjacent superior level at the final follow-up. Therefore, it is predicted that the disc of adjacent segment might initiate the degeneration [18–20]. In the short-term follow-up in our study, back pain could be quantified via VAS and ODI scores, and both scores in noninjury patients were lower than those in injury group. This result indicated that long-term therapeutic efficacy in noninjury group was better than that in injury group.

Physical conditions play an important role in the development of FV and ASD during MIS-TILF. Babu et al. [8] found that FV occurred frequently in younger patients less than 65 years old by retrospectively analyzing 279 patients who underwent minimally invasive surgery. They considered that the pedicle screw placement was affected in younger patients due to their stronger muscle. However, Park et al. [10] held the different point that FV had no correlation with age and gender. In our study, we found that obese patients were vulnerable to FV. A potential reason for this could be that puncture and Jamshidi needle move easily, which can damage the facet joint severely. Better anesthesia and fluoroscopy imaging techniques can potentially help avoid this damage in obese patients.

Most studies suggested that FV occurred in L4 and L5 more often than L1, L2, and L3. Anatomical studies confirm that the direction of facet joint articular surfaces transferred from sagittal to coronal plane in lumbar vertebrae [21]. In this study, among 29 patients with L5 top-screw level, FVs occurred in 17 cases (58.62%). In addition, the results showed that L5 was the most vulnerable segment. This could be due to the articular surface of L5 facet joint coronal presentation. In addition, the anterior lordosis curvature and thicker paravertebral muscles could potentially explain the increased FV at L4-L5.

Park et al. [10] found that the incidence of the FV was 50% (46/92) in patients and 31.5% (58/184) of the pedicle screws in these patients were placed by the percutaneous technique. In a review paper, Yson et al. [9] examined literature about open and percutaneous pedicle screw placement and found that screws of FV in open surgery was 183/803 (23%), but in minimally invasive surgery it was 94/354 (27%). In the present study, the results showed that FV's incidence was 39.39% (39/99) of the patients and 23.23% (46/198) of the screws in MIS-TLIF. The reasons of the different incidence of FV we analyzed are as follows: (1) In our minimally invasive insertion procedures, the screws were carefully placed to avoid deep penetration of the screw, so facet joint damage through the screw head penetration could be avoided [8]. (2) 3D-CT navigation was applied in this surgery to help navigate the screw insertion, which greatly reduces the damage of facet joints [9]. However, 3D-CT navigation system is too expensive for many hospitals and is difficult to set up initially. A more common practice is to use C-arm fluoroscopy in surgical procedures to clearly show the location of the pedicles according to the standard posteroanterior images in percutaneous pedicle screw placement technique.

This study is limited by several reasons. First, the overall sample size in this study is small. Specifically, the number of patients with grade 3 FV needs a larger sample group to reach a normal distribution and statistical reliability. The follow-up duration was short and radiographic methods were simple, which could potentially leave out explanations for our results. Lastly, biomechanical and kinematic studies should be done to determine the etiology of FVs.

## 5. Conclusions

We conclude that MIS-TLIF is a feasible technique to solve the lumbar disc degenerative disease effectively in lumbar spine, although FVs still occur commonly during follow-up and ultimately resulted in ASD. Future investigations regarding better surgical techniques based on MIS-TLIF need to be conducted to reduce the incidence of FV and consequent ASD.

## Figures and Tables

**Table 1:** General information of different FV patients in MIS-TLIF.

	Group A (31 patients)	Group B (22 patients)	*P*
*Age*	55.23 ± 12.09	52.23 ± 12.65	0.387
*Gender*			0.833
Male	15	10	
Female	16	12	
*BMI*	27.42 ± 1.83	28.51 ± 3.13	0.150
*Top-screw level*			0.828
L4	22	15	
L5	9	7	
*Follow-up time*	18.26 ± 3.78	18.59 ± 4.24	0.765

BMI: body mass index.

**Table 2:** Relationship between FV and relevant factors.

Factors	Patient (*N*)	Relationship between facet joint and patient		*P*
		Grade 1	Grade 2 + 3	
*Gender*				0.653
Male	51	32	19 (grade 2: 15, grade 3: 4)	
Female	48	28	20 (grade 2: 17, grade 3: 3)	
*Age*				0.566
<60	60	35	25 (grade 2: 21, grade 3: 4)	
>60	39	25	14 (grade 2: 11, grade 3: 3)	
*BMI*				<0.001^*∗*^
<30 kg/m^2^	62	46	16 (grade 2: 14, grade 3: 2)	
>30 kg/m^2^	37	14	23 (grade 2: 18, grade 3: 5)	
*Top-screw*				0.017^*∗*^
L3	4	4	0 (grade 2: 0, grade 3: 0)	
L4	66	44	22 (grade 2: 21, grade 3: 1)	
L5	29	12	17 (grade 2: 11, grade 3: 6)	
*Decompression*				0.864
Unilateral decompression	87	53	34 (grade 2: 27, grade 3: 7)	
Bilateral decompression	12	7	5 (grade 2: 5, grade 3: 0)	

BMI: body mass index; *∗* represents significant differences.

**Table 3:** Radiological and functional comparison between two groups.

	Group A	Group B	*P*
AIDH (cm)			
Preoperation	0.92 ± 0.28	0.92 ± 0.25	0.987
Final follow-up	1.10 ± 0.23^*∗*^	1.12 ± 0.19^*∗*^	0.797
Lumbar Cobb angle			
Preoperation	30.69 ± 11.60	33.32 ± 13.14	0.446
Final follow-up	31.79 ± 12.54	35.30 ± 9.22	0.269
Surgical Cobb angle			
Preoperation	14.13 ± 6.41	14.29 ± 6.63	0.931
Final follow-up	13.84 ± 5.76	15.05 ± 4.66	0.422
Low back VAS			
Preoperation	5.94 ± 1.03	6.23 ± 1.07	0.325
2-week postoperation	3.13 ± 0.56^*∗*^	3.23 ± 0.43^*∗*^	0.494
Final follow-up	1.48 ± 0.51^*∗*#^	2.32 ± 0.72^*∗*#^	<0.001
Leg VAS			
Preoperation	6.71 ± 1.01	6.86 ± 0.89	0.568
2-week postoperation	2.77 ± 0.62^*∗*^	2.86 ± 0.71^*∗*^	0.627
Final follow-up	1.45 ± 0.51^*∗*#^	1.50 ± 0.51^*∗*#^	0.734
ODI			
Preoperation	61.26 ± 11.17	57.73 ± 12.74	0.290
Final follow-up	15.06 ± 3.92^*∗*^	19.05 ± 5.30^*∗*^	0.003
ASAIDH (cm)			
3-day postoperation	1.07 ± 0.22	1.05 ± 0.23	0.730
Final follow-up	0.92 ± 0.23^*∗*^	0.91 ± 0.24^*∗*^	0.872
ASIDCA (°)			
3-day postoperation	7.07 ± 2.30	7.11 ± 2.78	0.955
Final follow-up	7.91 ± 2.63^*∗*^	8.76 ± 2.50^*∗*^	0.238

AIDH: average intervertebral disc height; VAS: visual analogue scale; ODI: Oswestry disability index; ASAIDH: adjacent superior average intervertebral disc height; ASIDCA: adjacent superior intervertebral disc Cobb angle. *∗* represents significant differences between the first observation point and the second or third observation point; # represents significant differences between the second observation point and the third observation point.
